# A systematic review of co-production approaches that involve family members, loved ones, or carers in the development of mental health or substance use resources/interventions

**DOI:** 10.1186/s40900-025-00758-4

**Published:** 2025-10-16

**Authors:** Jacqueline Carhoun, Raquel Nogueira-Arjona, Richard de Visser, Pablo Romero-Sanchiz

**Affiliations:** 1 School of Psychology, University of Sussex, Brighton, England; 2 Department of Primary Care & Public Heath, Brighton & Sussex Medical School, Brighton, England

**Keywords:** Co-production, Mental health, Substance use, Participatory research, Public engagement, Family involvement, Carer involvement, Public and patient involvement, Intervention development

## Abstract

**Introduction:**

The prevalence of mental health and substance use disorders is rising globally, significantly affecting not only individuals but also their families, loved ones, and carers. These affected loved ones are often overlooked despite providing unique insights that can contribute to the development of resources/interventions for these disorders. Co-production, a participatory approach in which public members are involved as equal partners in research, offers a valuable framework for including these individuals. However, involving affected loved ones presents specific challenges due to their emotional and vulnerable position.

**Methods:**

This systematic review aimed to address three questions: (1) What co-production procedures are used with affected loved ones in developing mental health and substance use resources/interventions? (2) How do researchers and stakeholders assess co-production experiences? (3) What components of co-production facilitate the involvement of the affected loved ones of those with mental health and substance use disorders or concerns? Eligible studies included at least one affected loved one in the co-production development of a mental health or substance use resource/intervention, provided a description of the co-production approach, and were in English. All dates were included in searches across seven databases using the Mixed Methods Appraisal tool and an adapted co-production checklist.

**Results:**

Content and thematic analyses were conducted, revealing three key themes for the meaningful involvement of affected loved ones in co-production: *creating a safe and trusting environment*, *facilitating accessibility and open dialogue*, and *integrating diverse perspectives*. Additionally, the review found variability in the use of co-production terminology.

**Discussion:**

These findings underscore the importance of prioritising inclusive, sensitive co-production approaches to ensure that the voices of those supporting individuals with mental health and substance use disorders are heard and valued. Future research should aim to clarify these terms and adopt standardised reporting styles to ensure sufficient detail and consistency in reports.

**Supplementary Information:**

The online version contains supplementary material available at 10.1186/s40900-025-00758-4.

## Abstract (plain language summary)

Mental health and substance use disorders are increasing worldwide, affecting not only individuals but also their families, loved ones, and carers. Despite their important role, these affected loved ones are often overlooked in research, even though their experiences could help shape resources and interventions. Co-production is a research approach that involves the public and views them equal research partners throughout a project. While this method can be valuable, involving affected loved ones in co-production can be challenging due to their emotional and vulnerable position.

This review explored how co-production has been used with affected loved ones to develop mental health and substance use resources or interventions. Specifically, it examined what co-production processes have been used, how the experiences of those involved have been assessed, and what factors make co-production effective.

The studies included in the review had to have at least one affected loved one in the co-production development of a mental health or substance use resource/intervention, provided details about the co-production approach, and were published in English. All dates of publication were included in searches across a variety of online databases. Two types of data analysis were applied, revealing three key themes for effective co-production: *creating a safe and trusting environment*,* facilitating accessibility and open dialogue*, and *integrating diverse perspectives.*

These findings demonstrate the importance of creating an inclusive and sensitive co-production space to ensure the voices affected loved ones are being heard and valued. Also, this review found differences in the wording used to describe co-production. Future research should work towards clearer definitions and standardized reporting guidelines to improve consistency in the field.

**Supplementary Information:**

The online version contains supplementary material available at 10.1186/s40900-025-00758-4.

## Introduction

Mental health and substance use disorders are increasing worldwide and are among the leading causes of disability [1], impacting not only the individual with the disorder, but also their families, loved ones, and carers. These disorders encompass a wide range of mental health concerns and symptoms that are diagnosable using standardized tools, such as the *Diagnostic and Statistical Manual of Mental Disorders*, 5th Edition (DSM-5) [2]. For consistency, this review will collectively refer to the disorders in the DSM-5 as “mental health and substance use.” The cascading impact of these disorders requires inclusive research to develop interventions (e.g. behavioural intervention) and resources (e.g. education platform) that provide meaningful and practical support [1, 3]. Supporting this, the World Health Organisation (WHO) 2013–2030 Mental Health Action Plan emphasised the need for a multi-faceted approach that involves the family members, informal carers, and significant others (now referred to as ‘affected loved ones’) in mental health research and service development [4]. The term ‘affected loved ones’ was chosen as ‘affected family members’ or AFM is widely used in the literature and many countries to represent family members impacted by their loved one’s mental health/substance use [5–8]. Therefore, to ensure this review’s reference included not only family members (e.g. mothers), but also other informal carers (e.g. person providing unpaid support), friends, and/or significant others (e.g. partner), this review has adopted the term ‘affected loved ones.’

Research shows that mental health and substance use disorders can significantly impact affected loved ones [9–11]. For example, family members of individuals with severe mental illness often experience psychological stress and socioeconomic challenges [10]. Accompanying the impact they face, the affected loved ones often maintain close emotional and physical proximity to those with mental health or substance use disorders, placing them in a unique position to contribute to treatment and recovery efforts. They can play an active role in the recovery process, offering emotional support, advocating for care, and managing day-to-day challenges. Their unique experiences and perspectives can enrich research and improve resources and interventions [12–16]. For instance, affected loved ones supporting an individual with a substance use disorder may have been directly involved in critical moments of the individual’s recovery plan, intervened during an overdose, or communicated with emergency and law services [13, 15]. Another study explored adult siblings of individuals with severe intellectual and developmental disabilities, identifying some key roles they engaged in like a legal representative or service coordinator [16]. These firsthand experiences provide invaluable insights that might be overlooked if research were limited to professionals or the individuals themselves. Their involvement in research could supplement developments for the individual with the mental health or substance use need, the affected loved one, or other resources/interventions that help support the targeted mental health/substance use disorder (e.g. mental health nursing curriculum outline).

Including affected loved ones in research not only broadens and deepens the scope of understanding of the problem, but also ensures that interventions are informed by those who have lived through the complexities of support and care [17]. Acknowledging their active role in the recovery process and integrating their contributions into research and service design are essential for developing inclusive and effective strategies to address mental health and substance use disorders. Despite the value of including the affected loved ones in research, a gap remains, most research focuses on the details of patient engagement (e.g. barriers) and the roles of family engagement are less considered and explored [18].

Involving affected loved ones offers benefits but requires acknowledging their emotional vulnerability. Furthermore, research suggests it is important to ensure the affected loved ones are well-represented to avoid the risk of them feeling tokenistic or overshadowed during the research process [19]. Therefore, research must be supportive, relevant, adaptable, and ethically balanced to prioritize their well-being alongside their contributions [19–21].

To ensure this, Public and Community Involvement and Engagement (PCIE) has emerged as a plausible element of inclusive research, being increasingly regarded as a gold standard in both research and healthcare development [22]. By actively involving the public as valued collaborators, PCIE moves beyond treating them as mere data sources, recognizing their contributions as integral to the research process. One form of PCIE, known as co-production, involves including the public as equal partners in the research design and execution [23]. Terms like co-design and co-creation are sometimes used interchangeably with co-production, as they all emphasize shared decision-making and active involvement at various stages of the research process. Some studies distinguish co-design as being specifically applied to the collaborative development of complex interventions [24, 25]. In contrast, co-creation is often described as generating new knowledge through collaboration between stakeholders and academics [24, 25]. Despite these distinctions, the literature often blurs the boundaries between these terms, as they all share a common principle: involving the public in power-sharing partnerships at various stages of the research process such as sharing knowledge, designing research features, and developing products [24, 25]. For clarity and consistency in this review, the term “co-production” will encompass the range of PCIE approaches that include the public throughout the research design and process.

Incorporating a co-production approach with the loved ones of individuals with mental health and substance use disorders can lead to more relevant and practical solutions to mitigate their impact [23]. Actively involving the affected loved ones at multiple stages of the research process ensures that resources/interventions are developed not only *for* them but also *with* them [22, 23]. This participatory process tailors resources/interventions to real-world needs and can foster a sense of ownership and connection among contributors [26]. Additionally, it may encourage altruism by letting them shape future services [27]. Involving the public and their unique perspectives helps ensure that research is not only responsive to their needs but also leads to ethical, sensitive, and relevant [26, 28].

Furthermore, other research and reviews have highlighted the benefits of public engagement in health, mental health, and substance use research [29–31]. For example, a recent scoping review demonstrated its ability to positively impact the public members and researchers’ experiences, and to also improve the quality and environment of the research [29].

Despite the benefits of co-production, it is essential to recognize its challenges. The process can be time-intensive and resource-heavy and may strain both stakeholders and researchers [32]. Additionally, differing perspectives may create power imbalances or tensions between researchers, professionals, and public participants, complicating collaboration [32, 33]. Due to the sensitivity of the affected loved ones of individuals with mental health or substance use disorders and to the challenges of co-production, it is essential to understand what factors contribute to the successful inclusion of this population in a co-production design. There is a current gap in the literature that seeks to understand this despite its importance in optimising an effective research environment. The aim of this review is to explore how affected loved ones are meaningfully involved in co-production processes when developing mental health or substance use resources and interventions.

### Objectives

This systematic review seeks to address this gap in the literature by evaluating existing studies and offering insights into best practices when developing mental health or substance use resources/interventions with affected loved ones. This includes examining the procedures, strategies, and experiences of affected loved ones in research to identify the most effective approaches. Evaluating the co-production process not only helps assess the outcomes of the developed interventions but also provides critical insights into how participants perceive their involvement. These insights can inform future practices and improve the design of studies that include affected loved ones in co-production. Moreover, by exploring how the terms “co-production” and “co-design” are applied and understood in practice, this review seeks to contribute to more consistent and effective use of terminology in mental health and substance use research. To address the above, this review seeks to answer three key research questions:

1. What procedures of co-production are used with the affected loved ones when developing a mental health/substance use resource/intervention?
2. How do researchers and other stakeholders (e.g. individuals coordinating the co-production) assess the co-production members’ experiences?
3. What components of co-production facilitate the meaningful involvement of affected loved ones?

## Methods

### Registration and protocol

The review protocol was registered in the International Prospective Register of Systematic Reviews (PROSPERO) under the ID CRD42024534533. This review followed the recommendations outlined in the Preferred Reporting Items for Systematic Reviews and Meta-Analyses (PRISMA) statement and adhered to the 27-item PRISMA checklist [34].

### Eligibility criteria

Studies were included if they utilised a co-production approach (or a related term) involving an affected loved one. At least one family member loved one (e.g., guardian, significant other, friend), or carer of an individual with a mental health need/disorder or substance use concern/disorder needed to be involved in the co-production process. Affected loved ones of all ages were included. Additionally, the co-production must have been used to develop or adapt a mental health or substance use resource/intervention. This adapted or developed resource/intervention did not only have to be for the affected loved ones, but it could support anything or anyone as long as it related to the targeted mental health and substance use issue. Studies were required to provide a description of how the co-production was utilised and had to be written or translated into English. There were no restrictions on the date of publication. Studies were excluded if they focused on other engagement terms that lacked the depth of involvement associated with co-production. For example, if a study involved individuals with lived experience at only one stage of the research process.

These criteria were applied during both the title/abstract screening and full-text review phases. Grey literature was included to reduce publication bias [35]. However, only empirical grey literature articles were collected to ensure evidence on the process and methods on developing the resources/intervention.

### Search strategy

A comprehensive systematic search was conducted in June 2024 and re-ran in January 2025. The search covered the Cochrane Central Register of Controlled Trials (CENTRAL), MEDLINE, Open Grey, ProQuest Theses and Dissertations, PsycINFO, Web of Science, and Scopus. Additionally, a secondary search was performed on Google Scholar, with relevant studies from the first 15 pages of results included in the screening process. Due to the large inclusion of results in Google Scholar, only the first 15 pages were reviewed to maximise relevance [36]. The search strategy included terms related to the target population of co-production approaches (e.g.co-creation), and mental health and substance use resources/interventions (e.g., treatment program), and affected loved ones. The term affected loved ones was operationalised using a combination of MeSH terms and keywords such as ‘brother’, ‘carer’, and ‘partner,’ to ensure comprehensive coverage of relevant literature. A full list of search terms is provided in Supplementary File 1. Search terms were required to appear in the title, abstract, or keywords to be considered for retrieval.

### Selection and data collection process

All retrieved articles were exported to Rayyan for systematic review [37]. After duplicate removal, the primary reviewer screened titles and abstracts, with the secondary reviewer independently screening 10% of the articles [38], selected at random to reduce bias. Full texts of relevant studies were retrieved, and the secondary reviewer randomly reviewed 10%. Regular meetings were held between the main reviewer and a second reviewer to ensure consistency in applying the assessment criteria. A senior reviewer was also consulted to provide oversight and methodological guidance throughout the appraisal process. Exclusion reasons were documented in Rayyan, and disagreements were resolved by consensus, involving a senior reviewer if necessary.

### Study quality and co-production assessment

The methodological quality of included studies was assessed using the Mixed Methods Appraisal Tool (MMAT), chosen for its ability to evaluate a variety of research designs [39]. To evaluate co-production quality, an adapted version of a participatory checklist [40] was used, tailored specifically to co-production by refining criteria. The adapted checklist included seven criteria assessing aspects like intent, setting, team composition, and phases, scored as “Yes” (1 point), “Limited” (0.5 points), or “No” (0 points).

The primary reviewer applied the MMAT and adapted checklist to all studies, while the secondary reviewer assessed 25% randomly. Discrepancies were resolved through consensus with a senior reviewer if needed. Scores were used to inform quality discussions but did not exclude studies Table 1.

**Table 1:** Adapted co-production checklist for sufficiency of reporting on co-production or related terms

Checklist item	Notes on item
1. Is the intention behind co-production terms described?	Y or N
2. Is there a description of the setting of co-production? Where/how did the co-production take place?	Y: provided details on the geographical location(s) and how the meetings/interactions took place (e.g. online meetings, focus groups). L: provided details of only one of these elements. N: not reported.
3. Is there a detailed description of the team members apart of the co-production approach?	Y: details on number of members *and* members described in more detail than “researchers”, “caregivers” (e.g. researcher’s expertise are noted, caregivers are categorised as family members, etc.) L: The numbers of members were described but details, not noted. N: not reported.
4. Is there a description of the resource/intervention that was developed/adapted provided?	Y: relevant details provided. L: missing some relevant details. N: not reported.
5. Are descriptions of the phases and methods of co-production provided?	Y: information on the methods used, phases (if relevant), and activities were reported. L: some but not all information provided. N: not reported.
6. Are descriptions of the intensity and schedule of the co-production approach provided?	Y: relevant details reported, e.g. how often the interactions occurred and for how long. L: missing some details. N: not reported.
7. Is the experience of the members involved in the co-production evaluated?	Yes or No

### Data extraction

The primary reviewer extracted data on study characteristics and co-production elements, recording variables in Excel, including author, year, country, study design, co-production terminology, mental health/substance use disorder/symptom, and developed/adapted resource/intervention. Notes were taken on team composition, frameworks, phases, participant compensation, and evaluation of members’ experiences (if reported). The above variables were chosen to understand the broader study characteristics (e.g. location) and to support the co-production checklist which considers the other elements as relevant to the co-production process. This extraction process informed both content and thematic analyses, as well as the adapted co-production checklist.

### Analysis

Two complementary analytical approaches were employed.

#### Reflexive thematic analysis

Reflexive Thematic Analysis (RTA) was applied selectively to studies that evaluated the experiences of co-production members. RTA was chosen for its flexibility and rigour in handling complex qualitative data, enabling meaningful findings without relying on a predetermined theoretical framework. It also emphasizes reflexivity, ensuring awareness of researcher biases and their impact on the data [41]. The RTA was informed by a constructivist epistemology to ensure the themes were regarded as interpretive concepts that were shaped through the researcher’s engagement with the data [42]. The selective application of RTA was only applied to included studies that evaluated direct participant feedback about their experiences on being a part of the co-production team. This approach was essential for identifying the components and strategies that enabled the involvement of affected loved ones in co-production, as perceived to be effective by those directly involved.

To conduct this analysis, the primary reviewer systematically coded the evaluations and co-production elements of the relevant studies. Themes were developed by categorizing and analysing the emerging patterns within the data. The coding and thematic development process utilized a combination of software tools (Excel and Zotero) and manual techniques (such as post it note organization) to facilitate a thorough and organised approach to theme development and data categorisation, while a reflexive journal documented the analysis process. To ensure rigour, regular team meetings were held to support a systematic approach to analysis. Discussions focused on ensuring that the themes accurately reflected the data and incorporated multiple researchers’ (JC, PRS, RNA, RdV) perspectives.

#### Relational content analysis

After conducting the RTA, Relational Content Analysis (RCA), also informed by constructivist epistemology, was applied in two phases across all included studies. RCA was selected for its ability to systematically explore both qualitative and quantitative data, offering a structured approach to identify connections and deeper insights across studies [43].

The first RCA phase focused on identifying and categorizing specific co-production details within each study, leading to the development of distinct co-production stages. Using an inductive approach, this analysis captured key characteristics and activities, providing a structured understanding of the co-production process across studies.

Following the initial RCA, a second phase explored how the co-production strategies and procedures identified in the studies supported the themes derived from the RTA. This RCA phase used a deductive approach, focusing specifically on strategies and relationships that related to the themes identified in the RTA of participant experiences. By re-examining the studies through the lens of these developed themes, this phase of RCA enabled a detailed analysis of how specific co-production practices contributed to the effectiveness themes reported by participants. This phase provided a relational mapping of co-production strategies to the RTA themes, deepening the analysis of effectiveness within the co-production context.

## Results

### Study selection

**Fig. 1:**
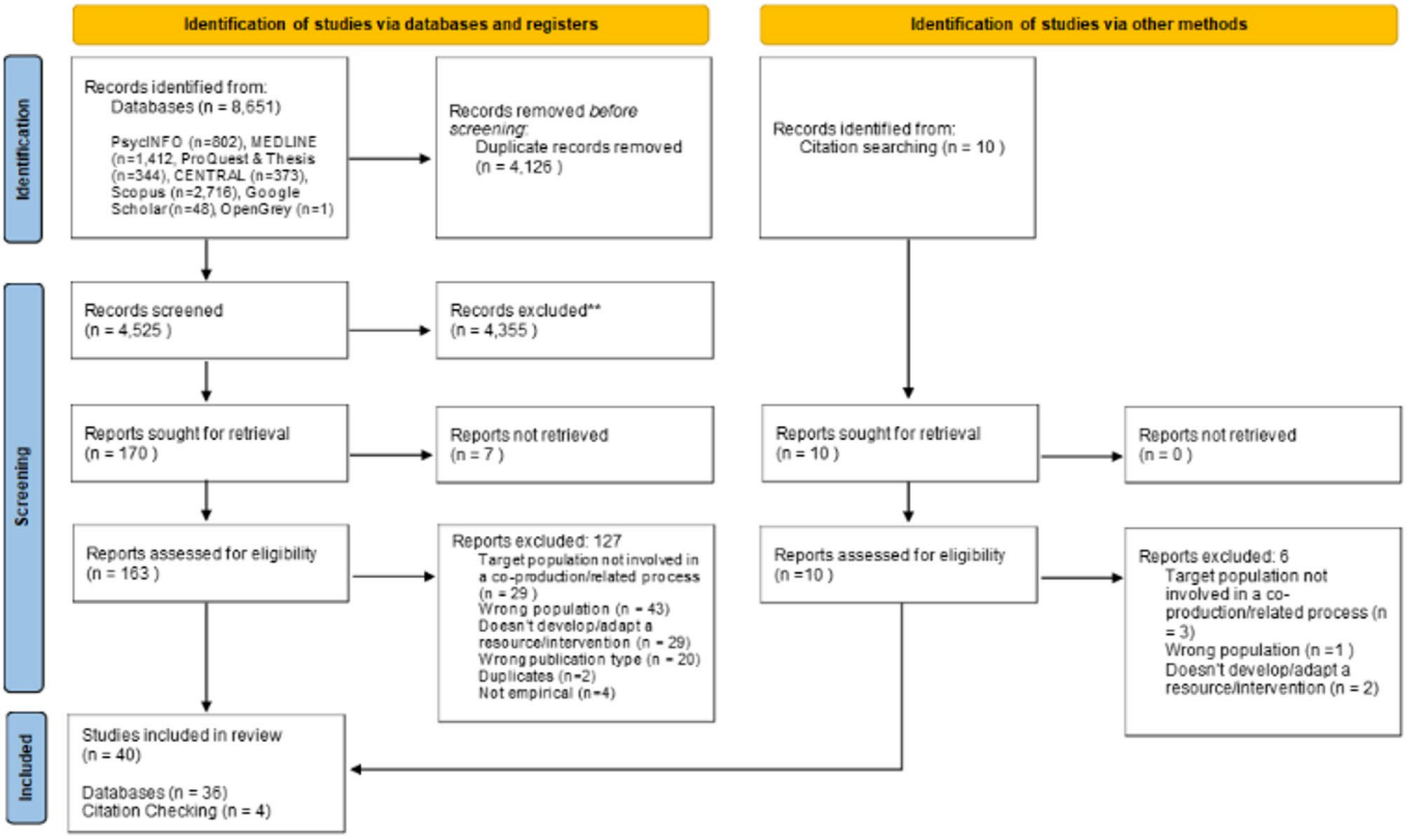
PRISMA 2020 Flow Diagram

The study selection process is summarised in the PRISMA flow diagram (Fig. 1). The articles were assessed based on their titles and abstracts, followed by full-text screening. Thirty-six articles met the inclusion criteria and were included in the review. Screening reference lists of the included articles identified four more articles. In total, 40 articles were included in this systematic review. One paper reported the process of two different co-production studies, only one of which met the inclusion criteria.

### Methodological quality

Overall, most studies reached medium to high methodological quality when assessed using the MMAT and the co-production checklist, with one study scoring in the low domain. (Supplementary File 2).

#### MMAT assessment

Twenty-three studies met all five MMAT criteria, with most being qualitative (*n* = 19) and the rest mixed methods (*n* = 4). One study, a mixed methods design, met two MMAT criteria.

The most common reason for not fully meeting MMAT criteria in qualitative studies was insufficient data reporting (*n* = 5). In the mixed methods studies, common issues included the absence of a clear rationale for employing a mixed methods approach (*n* = 5), failure to integrate qualitative and quantitative data (*n* = 4), and not interpreting the qualitative and quantitative findings together (*n* = 4).

#### Co-production quality assessment

Scores across the studies ranged from 3.5 to a perfect score of 7 mean = 5.13, median = 5, mode = 5; see supplementary file for table. Most studies scored well on the criterion assessing the intention: 34 clearly articulated their co-production rationale and goals. However, a considerable number of studies (*n* = 32) did not evaluate the experiences of the co-production team members, which contributed to lower overall scores.

### Study characteristics

In addition to addressing the research questions, we examined the characteristics of the 40 included studies (Supplementary File 3). Most studies were published after 2020 (*n* = 29), with others from the 2010s (*n* = 10) and the 2000s (*n* = 1). The majority originated from the UK (*n* = 16), Australia (*n* = 7), Canada (*n* = 6), and the USA (*n* = 2).

Methodologically, 27 studies were qualitative, and 13 used mixed methods. Various terms described the participatory engagement, including co-design, co-production, co-development, and co-creation.

The studies focused on loved ones supporting individuals with mental health or substance use concerns. The conditions included were dementia (*n* = 11), psychosis (*n* = 6), various mental health conditions (*n* = 5), autism (*n* = 5), severe mental illness (*n* = 4), intellectual/neurological disability (*n* = 4), eating disorders/disordered eating (*n* = 2), severe communication disability (*n* = 1), developmental disability (*n* = 1), and opioid use disorder (*n* = 1).

All included studies followed a co-production approach to develop a resource or intervention, with a variety of resources/interventions created or adapted. The most common types were interventions (*n* = 9), tool/tool kit (e.g. collection of resources or frameworks) (*n* = 5), or a digital platform/website (*n* = 5).

### Procedures and characteristics of co-production with affected loved ones

A relational content analysis of the 40 included studies identified the characteristics of co-production procedures used to engage affected loved ones in developing mental health and substance use resources/interventions, as detailed in Table 2.

#### Framework/approach

Most studies (*n* = 31) used an additional framework, theory, or approach alongside co-production, spanning 20 different frameworks. The most common were the Experience-Based Co-design Framework (*n* = 8), Participatory Action Research (*n* = 5), and the Behaviour Change Wheel Framework (*n* = 3). One study created its own integrated approach.

#### Co-production teams

Team size varied from 3 to 45 participants, with a mean of 18.6 and a median of 15. Some teams remained consistent throughout the study, while others experienced changes in composition during the co-production process. These changes typically occurred when participants left, were added, or were replaced. In some cases, new participants brought diverse backgrounds (e.g. professionals) and perspectives, while others had similar characteristics to those they replaced Twenty-nine studies reported such changes, while three did not specify team sizes.

The characteristics and roles of team members also varied. All studies included at least one affected loved one as part of their co-production efforts, however, the level of detail provided differed between studies. In addition to including affected loved ones, 36 studies incorporated other participants, with the most common being experts by experience or service users (*n* = 26), followed by academics and researchers (*n* = 21), and nurses and clinicians (*n* = 17).

#### Compensation

Most studies (*n* = 28) did not report how affected loved ones were compensated; emails were sent to these authors to obtain this information.

#### Stages and activities

An RCA identified five stages of co-production across the 40 studies, which were similar to those outlined by Freire and colleagues (2022). The stages the affected loved ones were involved in were sharing (*n* = 38), designing (*n* = 37), providing feedback (*n* = 25), evaluating (*n* = 16), and implementing (*n* = 5). Sharing involved participants contributing experiences, while designing allowed them to generate ideas for the resource/intervention. Feedback included suggestions for improvement, evaluation assessed the final product, and implementation involved administering the resource/intervention.

Various methods and activities were used across co-production stages. Co-production events, meetings, and workshops were the most common (*n* = 28), followed by focus groups (*n* = 20) and interviews (*n* = 20). The formats varied, with some conducted in person, others online, and a few unspecified. Additional methods included questionnaires and surveys (*n* = 7) and home observations (*n* = 1).

The most common activities were discussions and brainstorming, used in all studies (*n* = 40). Other frequently reported activities included art and writing exercises (*n* = 13) and voting, ranking, prioritizing, and rating tasks (*n* = 12).

**Table 2:** Co-production details in the included studies

Author, year	Framework/Approach used in co-production design	Members of co-production	Compensation for affected loved ones	Stages of co-production in which affected loved ones were engaged	Members throughout co-production	Data collection and engagement methods used with affected loved ones	Activities reported in co-production where affected loved ones participated
Acton et al., 2022 [44]	Systematic Adaptation Model	2 informal carers, 1 family member, 3 formal carers, team of academics, 2 occupational therapists, 1 nurse	Voluntary	Sharing, Designing, Providing Feedback	Same	In-person interviews, feedback questionnaires	Answering surveys/questionnaires, discussion
Brooks et al., 2021 [45]	Experience Based Co-Design	16 experts by experience, 7 parents/guardians, 2 academics, 9 other professionals	Gift or voucher	Sharing, Designing, Providing Feedback	Varied	In-person co-design events, in-person interviews, in-person focus groups, online focus groups	Art exercises, discussion, icebreaker activities, voting
Brooks et al., 2022 [46]	Intervention Development Process for complex Interventions, Experience Based Co-Design Approach	6 experts by experience, 9 carers/supporters, 5 professionals, 1 volunteer	Paid	Sharing, Providing Feedback	Same	Online focus groups, telephone interviews	Discussion
Cheng et al., 2024 [47]	User Centred Design	7 family caregivers	Gift card	Sharing, Designing, Providing Feedback, Evaluating	Same	In-person co-design events, in-person interviews	Answering surveys/questionnaires, brainstorming, discussion, thinking out loud
Chivers, 2005 [48]	Action Learning Approach	6 parents, 5 speech language pathologists	NR	Sharing, Designing	Varied	In-person co-creation workshops, in-person interviews	Discussion, mapping, storytelling
Cullingham et al., 2024 [49]	Experience Based Co-Design, Health Care Co-Design, Autism specific modules for Participatory Research	1–5 parents, 1–9 experts by experience, 2 clinical psychologists, 2 clinicians, psychiatrist, researchers	Paid	Sharing, Designing, Providing Feedback	Same	Interviews, online meetings	Discussion, iterative feedback processes
Davies et al., 2016 [50]	NR	Family carers, researchers, health care professionals (numbers NR)	Voluntary	Sharing, Designing	Varied	In-person interviews, in-person focus groups	Discussion, ranking, thinking out loud
Dodd et al., 2022 [51]	NR	2–12 experts by experience, 6 relative carers, ~ 6 spouses	Voucher	Sharing, Designing, Evaluating	Varied	In-person interviews, in-person meetings	Discussion, workbooks
Egan et al., 2023 [52]	NR	8 parents, 1 expert by experience, 1 researcher	Voucher	Sharing, Designing, Evaluating	Same	Online co-design workshops	Answering surveys/questionnaires, discussion, feedback loops
Goeman et al., 2017 [53]	Participatory Action Research	2–7 experts by experience, 1–22 carers, 1–8 nurses, 2 researchers, 1–2 clinicians, 20 healthy public members, 1–23 other professionals	Voluntary	Sharing, Designing	Varied	In-person and telephone interviews, in-person focus groups, in-person meetings	Discussion, mapping
Hackett et al., 2018 [54]	Experience Based Co-Design	12–19 experts by experience, 6–12 carers, 4–14 service providers	NR	Sharing, Designing, Providing Feedback	Varied	Interviews, phone application questionnaires	Brainstorming, developing touchpoints, discussion, mapping, ranking
Higgins et al., 2017 [55]	Participatory Action Research	Experts by experience, family members, range of clinicians, representatives from youth services, and academics (numbers not reported)	Paid	Sharing, Designing Providing Feedback, Implementing	Varied	In-person focus groups	Discussion
Jerwood, 2019 [56]	Developed own approach	7 experts by experience, 5 carers, 21 nurses, 3 support workers,12 clinicians, 5 therapists, 10 other professionals	Voluntary	Sharing, Designing	Varied	In-person co-design workshops, in-person interviews, in-person focus groups	Discussion, paper prototyping, prioritising exercises
Kaur et al., 2024 [57]	User-centred Design	1–2 experts by experience, 1–8 teachers/school coordinators/coaches, 3–4 parents, clinical psychologist, behaviour analyst	NR	Sharing, Designing, Providing Feedback, Evaluating	Varied	Online interviews, meetings, workshops, evaluation forms	Consensus building, discussion, rating, drawings,
Leadbitter et al., 2024 [58]	Experience Based Co-Design	Experts by experience 28 caregivers, 3 parents, therapeutic experts, third sector professionals	Voluntary/Vouchers	Sharing, Designing, Providing Feedback, Evaluating, Implementing	Varied	Focus groups, practice sessions	Discussion, mock sessions
Lopes et al., 2016 [59]	Participatory Action Research Methodology	1–11 experts by experience, 1–14 family carers	Voluntary	Sharing, Providing Feedback, Evaluating	Varied	In-person interviews, in-person focus groups	Brainstorming, discussion, reacting, using prototype
Mbazzi et al., 2020 [60]	‘Obuntu Bulamu’ Framework	~ 64 experts by experience, ~ 64 parents, ~ 33 teachers, other professionals, academics	Paid	Sharing, Designing, Providing Feedback, Implementing, Evaluating	Same	In-person focus groups, in-person meetings	Art exercises, discussion, filming, narrating, photos
McAllister et al., 2021 [61]	Experience Based-Co Design, Behaviour Change Wheel	15 experts by experience, 2 carers, 10 nurses, 4 care assistants, 3 clinicians, 1 student nurse	Voucher	Sharing, Designing, Providing Feedback	Varied	In-person co-design workshops, in-person meetings, telephone and in-person interviews	Affinity grouping, discussion, mapping, ranking, writing exercise
Middleton et al., 2023 [62]	Participatory Action Research Approach, Behaviour Change Wheel	3 experts by experience, 4 partner carers, 7 community providers, 7 researchers, exercise professionals	Voluntary	Sharing, Designing, Providing Feedback, Evaluating	Varied	Online and in-person focus groups, stakeholder workshops	Discussion, prioritising exercises, sharing exercises, thinking out loud, writing exercise
Milton et al., 2021 [63]	NR	13 experts by experience, 7 carers, 4 health professionals with lived experience, 21 health professionals	Voucher	Sharing, Designing, Evaluating	Varied	Participatory design workshops	Creating personas, discussion, art exercises, prototyping exercises
Molloy et al., 2024 [64]	Design Thinking Approach	1 expert by experience, 2 carers, 2 nurses	Paid	Sharing, Designing, Providing Feedback	Same	Focus groups	Discussion, photos
Mulvale et al., 2021 [65]	Experience Based Co-Design, Continuity Vortex Model	9–12 experts by experience, 7–8 carers, 8–10 service providers	Voluntary but received honorariums	Sharing, Designing, Providing Feedback	Varied	Co-design event, in-person and online focus groups	Answering surveys/questionnaires, brainstorming, sharing exercises, videos
Murfield et al., 2022 [66]	Person-based Approach	6 family caregivers	Paid	Sharing, Designing, Providing Feedback	Same	Online, telephone, and in-person interviews, online co-design sessions	Creating scenarios, creating personas, discussion, videos
Nakarada-Kordic et al., 2017 [67]	NR	Experts with experience, family members, clinical staff, researcher, designer (numbers NR)	Voucher	Sharing, Designing, Providing Feedback	Varied	In-person co-design workshops	Art exercises, brainstorming, card sorting exercise, ice breaker activities, mapping, ranking, storytelling
Oksnebjerg et al., 2019 [68]	NR	4–14 experts with experience, 2–4 family carers, 4–6 formal carers	NR	Sharing, Designing	Varied	In-person interviews, in-person focus groups, in-person workshops	Discussion, poster presentations, using protoype
Oostra et al., 2023 [69]	Human Centred Design Approach	3–7 informal carers, 1–2 nurses, 2–4 other professionals	Voluntary	Sharing, Designing	Varied	Co-creation meetings	Defining ‘point of focus’, discussion, consensus exercises
Rapaport et al., 2018 [70]	NR	4–5 carers, other professionals	Paid and Voucher	Designing	Varied	In-person co-production meetings, in-person and online focus groups	Discussion
Rathnayake et al., 2021 [71]	Adult Learning Theory	2-166 carers, 1–7 professionals (researchers, nurses, clinicians), IT experts and designers	NR	Sharing, Designing	Varied	In-person interviews, online surveys	Answering surveys/questionnaires, discussion, ranking
Rivard et al., 2024 [72]	Community Based Participatory Approach	24 stakeholders (parents, health professionals, administrators)	Gift cards	Sharing, Designing, Evaluating	Varied	In-person interviews, in-person focus groups	Answering surveys/questionnaires, discussion, validation and feedback exercises
Robinson et al., 2020 [73]	NR	2 parents,1 expert by experience, 1 researcher	NR	Sharing, Designing, Implementing, Evaluating	Same	In-person interviews, home observations, in-person meetings	Diary recording, discussion
Sin et al., 2019 [74]	Resource Development Cycle Framework	3 experts by experience, 3–24 family carers,1–2 clinicans,1 researcher, 1–3 other professionals	Paid	Sharing, Designing, Providing Feedback	Same	Expert advisory group workshops, in-person focus groups	Art exercises, discussion, prototyping cycles, using prototype, videos
Tarver et al., 2021 [75]	NR	4 experts with experience, 5 carers/parents, 5 teachers, 5 clinicians	NR	Sharing, Providing Feedback, Evaluating	Same	In-person focus groups	Answering surveys/questionnaires, discussion
Turuba et al., 2024 [76]	Design Thinking Approach, Community Based Participatory Research	12 carers, 1 support worker, 1 project lead	Paid	Sharing, Designing, Providing Feedback, Implementing, Evaluating	Same	Online co-design meetings	Answering surveys/questionnaires, brainstorming, discussion, prioritising, writing exercises
Ung et al., 2023 [77]	Delphi Approach	2–4 experts by experience/carers, 2 researchers, 1 psychiatrist, 1 academic, 1 counsellor	Paid	Sharing, Designing, Providing Feedback	Varied	Co-design meetings, online surveys	Answering surveys/questionnaires, discussion, creating scenarios, rating
Vijayalakshmi et al., 2024 [78]	Experience Based Co-Design	4–9 carers, 4–9 experts by experience, 2–16 mental health professionals, 2 physical trainers	NR	Sharing, Designing, Evaluating	Varied	In-person co-design conferences/workshops, in-person interviews, in-person focus groups	Developing touchpoints, discussion
Wittich et al., 2023 [79]	Consolidated Framework for Advancing Implementation Science	9 researchers, 3 informal caregivers, 3 members of Alzheimer’s Society (all with lived experience)	Voluntary or worked during working hours (paid by usual working contract)	Designing, Providing Feedback	Same	Online focus groups	Discussion, homework
Wood et al., 2023 [80]	Intervention Development Process for Complex Interventions Design	2 experts by experience, 1 expert by experience researchers, 2 carers, 2 clinical psychologists, 1 occupational therapist, 1 nurse, 1 assistant psychologist/researcher	Paid	Sharing, Designing	Same	Online co-production meetings	Discussion, summaries
Wormdahl et al., 2022 [81]	Participatory Action Research, Template for Intervention Description and Replication	1–3 carers,9 experts by experience, 77 health professionals,12 other professionals, 4 researchers, 5 students,	Voluntary, compensated for travel and loss of income	Sharing, Designing, Providing Feedback	Varied	In-person conferences, in-person and online meetings	Brainstorming, discussion, feedback loops, mapping, poster sharing, prioritising, voting
Zervogianni et al., 2020 [82]	Delphi Approach	5–6 experts by experience, 8 − 2 family members, 11–12 researchers, 13 professionals	Voucher	Sharing, Designing, Providing Feedback, Evaluating	Varied	Online surveys	Brainstorming, discussion, matching, ranking, rating, using prototype
Zhu et al., 2024 [83]	Behaviour Change Wheel, Theoretical Domains Framework	12 experts by experience, 4 family carers, 5 therapists	Voluntary	Sharing, Designing, Providing Feedback, Evaluating	Varied	In-person co-design workshops	Art exercises, brainstorming, discussion, prioritising, using prototype, voting

Note. Terms reflect the language used in each study and may differ from those used in the search strategy

### Methods of evaluating co-production team members’ experiences

Out of the 40 included studies, only eight recorded evaluations of members’ experiences involved in the co-production process. Of these eight studies, four used a single method of evaluation [48, 64, 72, 76], two employed multiple methods [62, 65], and two did not specify the evaluation method [61, 80]. The most common evaluation method was the use of self-report surveys or individual interviews [62, 65, 72, 76], administered throughout the co-production process [65], at the mid-point [48, 76], and at the end [48, 62, 72, 76]. Molloy and colleagues (2024) dedicated their final focus group to exploring members’ experiences within the co-production process. Other studies collected feedback continuously [62] or conducted evaluation interviews at midpoints and conclusions [48, 65].

### Components to facilitate the involvement of affected loved ones in co-production

To assess what components facilitated the involvement of affected loved ones in co-production, we analysed the findings of the eight studies that evaluated their experiences using RTA. Although these studies did not always distinguish between the contributions of affected loved ones and other co-production team members (e.g. clinicians), the themes identified still offer valuable insights for future research specifically involving the affected loved ones. The three key themes identified as particularly effective were *creating a safe and trusting environment*, *facilitating accessibility and an open dialogue*, and *integrating diverse perspectives* (Table 3).

The other 32 included studies did not evaluate the experiences of their co-production teams and were not included in the RTA. However, an RCA revealed that many of their approaches and strategies reflected the same emerging themes.

#### Creating a safe and trusting environment

This theme was crucial for enabling affected loved ones to feel secure, respected, and confident in their participation. Seven of the eight studies that reported evaluations contributed to this theme [48, 61, 62, 64, 65, 76, 80]. Participants in these studies reported feeling safe and supported which facilitated open communication [61, 62, 76, 80]. Additionally, trusting relationships and feelings of empowerment within teams were commonly reported [48, 64, 65]. However, one study noted a lower score on a self-report survey in this area, with an individual feeling disconnected from the team; the authors state this is possibly due to high dropout and turnover rates of team members [62].

Various strategies were employed to *create a safe and trusting environment*. Many studies dedicated their initial meetings or focus groups to building relationships, establishing ground rules, and setting safety and confidentiality expectations [61, 62, 64, 76, 80]. Some studies revisited these expectations regularly, beginning subsequent meetings with reminders about safety and confidentiality [76, 80] or reaffirming team values [62]. Breaking down larger groups into smaller ones to enhance trust and bonding was also a common strategy [61, 65, 76]. In one study, ensuring all members had an opportunity to speak was a key method to enhance participation [80].

Among the 32 studies that did not evaluate their co-production experiences, 16 employed strategies encompassing the theme of *creating a safe and trusting environment*. Specifically, other studies dedicated their initial meetings to relationship-building and agreeing on core values (*n* = 4) and used smaller working groups with members of similar experiences (*n* = 11). Additionally, various studies did not record the discussions or design events with the co-production members to ensure they felt comfortable and to create an authentic environment (*n* = 4). Other strategies that support the theme reflected debriefing the affected loved ones and offering support after the sessions (*n* = 2), including local advocacy members with lived experience to reduce any power imbalances (*n* = 1), and fostering a welcoming atmosphere by providing snacks, refreshments, and adopting a casual dress code (*n* = 2). Another study encouraged the researchers to become immersed in the culture of the members they were working alongside (*n* = 1).

#### Facilitating an accessible and open dialogue

This finding emerged as a compelling theme, highlighting the importance of accessible tools and strategies that promote meaningful discussions. This theme was present in five studies that evaluated co-production experiences [62, 64, 72, 76, 80]. Participants valued models that were easy to follow and understand [62, 72, 76], appreciated regular feedback on the design process and felt well-informed throughout [80]. Effective organisation and use of tools were also reported to facilitate productive discussions [64, 76].

The strategies linked to this theme included providing relevant research, summaries, and webinars to team members [62, 76], training project leads in methodology and group facilitation [76] and offering early access to materials [62, 72]. Some studies organised activities to encourage discussion by providing pre-agenda discussion questions or offering regular feedback from previous meetings [64, 80].

From the studies that did not evaluate the experiences, RCA identified strategies that reflected this theme in 27 of these 32 studies. *Facilitating accessibility and open dialogue* was supported by researchers presenting background and relevant information to the team members (*n* = 12), training some or all members in relevant skills (*n* = 3), and providing the team with early access to materials (*n* = 3). Also, multiple studies provided organised activities (*n* = 16) and presented discussion prompts (*n* = 12) like pre-developed questions (*n* = 2), topic guides (*n* = 5), feedback loops (*n* = 1), touchpoints (*n* = 2), and structured agendas (*n* = 3) to the team members.

#### Integrating diverse perspectives

This last theme emphasised the importance of including and recognising the varied viewpoints of all stakeholders, including the affected loved ones. Four of the eight studies evaluated participant experiences contributing to this theme [48, 64, 65, 76]. Participants reported that incorporating multiple perspectives helped them connect with and understand different viewpoints, leading to a more comprehensive approach to co-production [48, 65]. Sharing perspectives also clarified their thinking and reinforced their ideas [48, 64, 65]. However, one study highlighted the group’s perceived lack of diversity, noting that most members were white, female, and middle-class [76].

Strategies employed by these studies to integrate diverse perspectives included assembling diverse co-production teams, including affected loved ones, service users, academics, and professionals [48, 64, 65, 76]. In three studies, teams were divided into smaller working groups to ensure that various perspectives were represented at different stages [48, 65, 76]. Activities designed to share and integrate perspectives, such as carousel-sharing or consensus-building exercises, were also utilised [48, 65]. One study acknowledged the challenges of differing perspectives but emphasised the importance of including diverse viewpoints [64].

Of the studies that did not evaluate the experiences, RCA identified 28 incorporated the theme of *integrating diverse perspectives* into their co-production design. These studies included diverse team members (*n* = 28), though in three, members worked individually or with similar experiences and did not collaborate. One study reported a diverse team but noted that professionals had a significant influence, and more public members were needed. Some studies used smaller working groups (*n* = 12) to ensure diverse perspectives, and four used activities to encourage perspective sharing.

**Table 3:** Effective themes when including an affected loved one in a co-production approach to developing a mental health and substance use resource/intervention

Theme	Sub Themes	Examples from text
Creating a safe and trusting environment.	Relationship building Confidentiality Welcoming environment	“The group supported one another and checked-in with one another after difficult discussions, which contributed to the group being a safe space. This was something the group thought went well throughout the coproduction process, which was due to the establishment of meaningful and respectful relationships” ([80], p. 10). “A key to understanding the group’s sense of a successful collaboration was the development of trusting relationships; they respected each other’s position and enjoyed each other’s company” ([64], p. 307).
Facilitating accessibility and an open dialogue.	Structured and organised engagement Functional and comprehensive technology and materials Inclusive activities	“Caregivers also highlighted the impact of good facilitation skills by the co-leads, which resulted in meetings described as well-organized, clear, and well-paced” ([76] p. 8). “All respondents also agreed that they found the technology was easy to use for the purpose of the [co-production] process and was reliable” ([62], p. 1667). Evaluations of experiences mentioned that “another aspect that went well was the regular feedback about how the groups contributions had shaped the intervention development” ([70], p. 10).
Integrating diverse perspectives.	New perspectives and understanding Importance of diversity	“A youth described gaining ‘new understanding [of] what my mom went through,’ and being ‘brutally honest’ with other caregivers, in a way that they could not be with their own mother” ([65], p. 153). During an end-point survey after the co-production process, “caregivers described missing perspectives from people of color, new immigrants, fathers, grandparents, and foster parents” ([76], p. 8).

## Discussion

The 40 included studies underscore the growing importance of involving affected loved ones in the co-production of mental health and substance use resources/interventions. This review highlights the essential components to facilitate involving the affected loved ones and how the field of co-production research can be strengthened.

### Strategies to facilitate the involvement of affected loved ones in co-production

*Establishing a safe and trusting environment* is essential when involving the loved ones of those with mental health and substance use disorders in co-production. Participants emphasised the importance of feeling valued and supported, and strategies such as fostering confidential and inclusive spaces were instrumental in achieving this. The literature highlights the importance of building relationships and trust to foster a successful research environment [84–87]. Wilkins [87] states that involving the public as research partners, rather than just participants, requires establishing trust and respectful relationships, as these stakeholders may lack human research protections (e.g. ethical approval) compared to traditional participants. Moreover, stakeholders often navigate unfamiliar research settings, leaving them more vulnerable and reliant on researchers for guidance, resources, and leadership [87]. Similar findings have been found in other reviews that explored public engagement in mental health and substance use [29, 88]. For example, a scoping review highlighted that feeling accepted, building trust, and developing strong relationships helped facilitate impactful engagement [29]. Creating a safe and trusting environment is particularly crucial given the unique stigma, power, and uncertainty that often surround these disorders [89].

A National Institute for Health and Care Research funded programme developed a co-production guide that also underscores the importance of a supportive research environment through engaging in shared decision making, a focus on relationship building and respecting everyone’s experiences and contributions [23]. Similar values are found in the Ottawa Patient Engagement in Research Model, which emphasises inclusiveness, support, mutual respect, and partnership [86].This guide and model, developed in different countries, supports our finding by highlighting the importance of a comfortable research environment when involving the public in research. Future co-production studies must prioritize these principles to ensure a comfortable and supportive research environment for the affected loved ones.

*Facilitating accessibility and an open dialogue* to engage affected loved ones in a co-production approach is another important strategy. Many studies took action to enhance accessibility and encourage meaningful participation, which aligns with findings from Harrison’s review [85]. Their narrative review identified training, education, and bidirectional communication as common best practices for engaging patient stakeholders, including families and caregivers, in research across 55 studies [85]. This is particularly important for affected loved ones who may not be familiar with research settings or formal communication methods, such as research frameworks, terminology, or expectations during focus groups or interviews. To address this, Concannon and colleagues [90] emphasise the importance of developing an engagement plan before beginning stakeholder research to ensure consistent and meaningful involvement. The current review similarly recommends that co-production studies involving affected loved ones develop a detailed engagement plan. Such a plan should address areas like stakeholder training, education, accessibility, and specific activities designed to create an approachable research environment. However, facilitating this kind of environment can be time consuming and costly to researchers. To minimise this, researchers can ask the co-production team members what training they require and ask them to contribute ideas to developing activities. By outlining these considerations, researchers can ensure that affected loved ones feel comfortable, empowered, and actively engaged, ultimately leading to richer, more meaningful contributions to the research process.

Another strategy to consider is *integrating diverse perspectives*. The inclusion of diverse stakeholders is a foundational principle in patient engagement research and is considered important for its success [22, 85]. Diversity within the co-production teams is vital, as affected loved ones often bring different experiences, such as providing support in the home, than professionals or service users who are more directly involved in mental health or substance use services. These varied experiences provide valuable perspectives that enrich the collective understanding of the issues at hand [91]. Moreover, one of the core principles of co-production is ensuring public involvement in research teams to guarantee diverse input [22, 23]. Failing to prioritize diversity risks limiting the breadth of perspectives gathered and undermines the core objectives of co-production. However, with an increased number of perspectives there is potential for an increase in differing opinions which could lead to conflict or challenges amongst the team. Researchers must be aware of this and prepare to diffuse the situation. For example, they could prepare a guiding phrase to end certain conversations or engage in a break when the challenge arises. Therefore, building diverse co-production teams is not only beneficial but necessary to achieve the full potential of co-production, however, the researcher must prepare for potential challenges.

These themes are evident across the co-production procedures in the included studies. Many studies incorporated an additional framework, such as Design Thinking, alongside co-production. Design Thinking is a dynamic, collaborative, and structured 5-step approach to problem-solving that balances flexibility and organization [92]. Incorporating such frameworks can enhance accessibility within co-production teams by providing structure. However, it is crucial to select frameworks carefully, ensuring they complement the iterative and adaptive nature of co-production [23]. Striking a balance between structure and flexibility is vital for *facilitating accessibility and an open dialogue* and ensuring the needs and contributions of loved ones are valued.

These findings go beyond thematic insight and highlight practical strategies that can be integrated into the design and implementation of co-production. Creating a safe and trusting environment, facilitating accessibility and open dialogue, and integrating diverse perspectives are not only foundational principles of effective co-production, but can also function as intervention strategies when intentionally embedded into the research process. For example, some included studies provided training and pre-meeting briefings to affected loved ones, which supported both comfort and participation [61, 76]. These approaches can inform future studies aiming to develop more inclusive, responsive, and effective mental health and substance use interventions in partnership with affected loved ones.

Furthermore, this review highlighted an important finding regarding the involvement of affected loved ones in different co-production stages. The five identified stages in the included studies showed a decrease in the frequency of affected loved ones’ engagement, suggesting their involvement tapered off as the research progressed. Including the affected loved ones in more stages will require time, funding, and resource demands [32, 93, 94]. However, it is possible to encourage the affected loved ones’ participation in other stages by implementing feedback using other accessible tools like email or phone calls to increase accessibility [62, 65, 71]. To encompass the ideology of co-production and to maximise collaboration with the affected loved ones, this review suggests incorporating the affected loved ones in as many stages as possible. This is important because excluding the affected loved ones from late stages-such as implementation and testing- could negatively influence the design’s effectiveness and the product’s practicality. However, this review recognizes the previously mentioned challenges associated with increasing participation at the later stages, and recommends finding a balance of engagement and resource expenditure. Including affected loved ones in multiple stages makes the co-production process more comprehensive, allowing for the *integration of diverse perspectives* at every stage.

Our review included both studies where affected loved ones were directly involved in co-production based on their own experiences, and studies where they contributed as proxies on behalf of individuals with mental health or substance use disorders. This distinction highlights an important variation on how the affected loved one’s involvement is operationalised. The findings of this review, particularly those related to the three themes, are relevant to both contexts. However, proxy participation also raises unique ethical and methodological considerations that may require tailored engagement strategies in future work.

Only eight studies assessed co-production team members’ experiences, aligning with calls for more research in this area [94–97]. Knowledge barriers, such as unclear terminology, can hinder communication and engagement if addressed too late [98]. Regular assessments help identify and resolve issues early, fostering inclusivity and improving research quality [32]. While all evaluations provided insights, this review suggests that ongoing or mid-point feedback is more effective. However, it is important to ensure the co-production team members’ still feel they are valued research team members, not only sources of data collection. If the evaluations of their experiences are not too extensive, this approach may ensure participants feel valued and comfortable by *creating a safe and trusting environment.*

### Enhancing co-production research: reporting, inclusivity, and methodological rigour

Established guidelines, such as the Guidance for Reporting Intervention Development Studies (GUIDED) (80) and the Guidance for Reporting Involvement of Patients and the Public (GRIPP2) (81), play a crucial role in improving research quality. The use of these guidelines promote transparency, enhance reproducibility, and provide structured frameworks that help researchers report their methods in a clear and rigorous manner. GUIDED and GRIPP2 are evidence-based guidelines to ensure that studies involving intervention development and the public, report enough information to ensure the quality of reporting is reached. However, literature suggests that these guidelines need to be considered more as a guide than a concrete checklist, particularly in mental health and substance use research because of the complexities of differing stigma and power imbalances often associated [89].

Despite co-production guidelines in the literature [23], this review highlights the need for a structured co-production reporting process for researchers to follow. This review suggests future research to develop a specific co-production reporting guideline to address the varying reporting styles and documentation of co-production. Such a guideline would help standardize the reporting of co-production methods, ensuring they are applied and reported with rigour. Additionally, co-production studies could be pre-registered to support the reporting and documentation of co-production practices.

The included studies mentioned various co-production-like terms (e.g. co-development). However, the terms were indistinctive, and no specific patterns or differences were found among the studies. The literature also has varying results regarding the differences and similarities between the terms. Some definitions suggest that co-production involves various stakeholders throughout the project and that co-design is mainly used in developing complex interventions [24, 95]. However, the characteristics from this review and some other literature suggest that the terms are more widely used interchangeably and can incorporate a variety of goals and procedures along the way that encompass the larger picture of including stakeholders throughout multiple stages of a research design [22, 24, 95]. This interchangeable use of terminology reflects a broader challenge in the field, where a lack of consistency may hinder the development of standardised practices. The inconsistency in terminology is not novel to general engagement research, particularly surrounding mental health and substance use [99], supporting our review’s recommendation of adopting clearer definitions. Therefore, co-production can be defined as the collaboration of researchers, practitioners, and public members as equal partners throughout various stages of the research process [23]. It prioritizes shared power, mutual respect, and diverse expertise to develop and refine practical solutions. Adopting a cohesive definition and establishing standardized international guidelines could enhance the understanding of co-production in the literature, clarify its similarities and differences, and lead to more reproducible approaches and studies.

The need for developing standardised reporting and guidelines might be reflected in the finding that most studies were published in the 2020s, reflecting a potentially more recent field. Furthermore, this finding suggests that including this population is a novel approach when incorporating the affected loved ones into mental health development and design. Despite the WHO’s global plan to include the affected loved ones in mental health and substance use development, most included studies were conducted in high-income countries. This reflects a challenge in co-production research as funding, time, and resources can be seen as a barrier to effective approaches [32, 94]. The socio-economic and cultural backgrounds of participants also play a critical role in co-production research. Individuals from lower socio-economic backgrounds often face barriers such as financial constraints, stigma, and limited access to education, which hinder their ability to participate in these initiatives [100]. This may further limit the knowledge and use of co-production terminologies and strategies. Another contributing factor could be variations in terminology; co-production and PCIE may be described differently across contexts, affecting how such initiatives are understood and adopted globally. This factor may contribute to the explanation of why many included studies originated from the UK, perhaps this term is more widely recognised and adopted in the UK’s context. Future research should explore the adoption of the term ‘co-production’ across countries and prioritise inclusivity by exploring and addressing these regional and socio-economic differences, ensuring that diverse voices, particularly those of affected individuals and their loved ones, are represented in co-production worldwide.

While the geographic trends and policies highlighted a gap in the literature, the targets of the resources/interventions developed in the included studies demonstrated a need for further attention. Most studies developed a product designed for individuals with mental health disorders. While only a third of the studies specifically developed resources, (e.g. self-compassion intervention for family carers) [66], to support the affected loved ones. This finding highlights the lack of support developed for the affected loved ones who are involved with an individual with mental health or substance use challenges. These gaps have also been shown in the literature [101, 102]. A systematic review of carers of those who have been detained for mental health issues discovered that there was a lack of support for the carers at multiple time points, leaving them with overwhelming feelings regarding care and responsibility [102]. In the future, more research must include the affected loved ones in a co-production approach to develop resources specifically tailored to their needs.

To adhere to the principle of co-production, which regards the public members of the team as equal and contributing researchers, this review states that it is important to compensate the affected loved ones for their time and involvement in the co-production research. Compensation can help create a more diverse group of affected loved ones, as individuals who may otherwise face financial or logistical barriers—such as travel costs or childcare—can participate, highlighting one of the key themes of this review. Additionally, compensating team members conveys appreciation and respect for their time and perspectives [103]. Most studies did not report whether affected loved ones were compensated, making it difficult to assess participants’ motivations. Without transparency, readers may speculate whether participation was driven by personal connection or financial incentives. This is crucial, as compensation can influence demographics—higher pay may attract lower-income individuals, while unpaid roles may appeal to those with higher incomes [104, 105]. Failing to disclose this information risks unnoticed bias, affecting study interpretation. Transparent reporting of compensation ensures accurate, responsible, and reproducible co-production research.

This review demonstrates the involvement of loved ones is crucial across disorders, yet this review highlights a gap: only one study addresses substance use disorders. Turuba and colleagues developed a handbook to support parents and carers of youths with opioid use disorder, illustrating the value of including loved ones in this research [76]. The inclusion of only one study on substance use disorders, especially during an ongoing opioid crisis, is concerning, given the heavy burden on loved ones from stigma, guilt, and a lack of support [106–108]. Literature consistently highlights the integral role of loved ones in the day-to-day management and recovery process [108–110]. This gap may stem from the stigma and criminalization associated with substance use [111, 112] which can hinder research and advocacy efforts.

While most studies showed medium to high methodological quality, they also revealed areas for improvement. Some mixed-methods studies successfully incorporate both qualitative and quantitative approaches, demonstrating the value of this design by contributing to a more comprehensive understanding of their topics. However, many mixed-methods studies lacked a clear rationale for combining qualitative and quantitative approaches or did not fully integrate their findings. This challenge aligns with previous literature [113–115]. For instance, Younas [115] found that 31% of mixed-methods studies in nursing did not justify their choice of design. A clear rationale is essential for ensuring that mixed-methods research offers a cohesive understanding, rather than appearing as separate qualitative and quantitative studies [116]. Additionally, stronger integration of findings would deepen the research and potential impact of the mixed methods design by uncovering key connections [116, 117].

Majority of qualitative studies provided tables and quotes which strengthened the transparency and understandings of the findings. However, some qualitative studies did not include raw data, such as verbatim quotes, which can allow readers to better assess how conclusions were derived (79). To address the above, future studies should ensure that mixed methods research includes a clear rationale and better integration of findings. When integration is not feasible, researchers might consider publishing separate studies for each method to maintain methodological rigor. Additionally, to promote transparency, qualitative studies should consider including raw data [118].

The co-production checklist highlighted that some of the studies adequately reported the co-production elements, which is important to contextualise the findings and replicate the studies. However, some studies did not report this in enough detail. For example, many studies did not detail the composition of their co-production teams. This can hinder the readers’ understanding of how the team’s dynamics influence interpretation, an important aspect of co-production [23]. The checklist also identified that many studies need to document the intensity and scheduling of co-production activities; this is essential to ensure that procedures can be replicated for future studies.

### Limitations

Despite this review’s findings, several limitations must be acknowledged. First, this review exclusively focused on mental health and substance use, which may limit its generalisability to other health conditions or psychosocial challenges. Expanding the scope to include additional health concerns may have provided a more comprehensive understanding of how affected loved ones contribute across diverse contexts. Such inclusion might reveal different experiences and co-production dynamics, offering richer insights for researchers working with broader populations. However, we consider the specificities of mental health/substance use disorders merit a separate review to underscore the different needs of the affected loved ones in co-production.

Furthermore, the inclusion of only English-language studies, potentially introduces a cultural bias. Excluding non-English literature may overlook diverse perspectives on mental disorders and the experiences of affected loved ones, limiting the findings’ broader applicability. Future studies should consider addressing this potential bias by expanding search terms and inclusion criteria to encompass a broader range of culturally specific terminology and languages related to mental health and substance use disorders.

Moreover, the review concentrated specifically on the co-production phase of the studies without including other phases of larger-scale research where co-production may have been only one component. This may overlook the broader context in which co-production occurs, potentially limiting the ability to fully understand the impact and integration of co-production within multi-phase research projects. Future reviews that include the entire research lifecycle, from planning to implementation and evaluation, would provide a more holistic perspective and contribute to a deeper understanding of the role of co-production in study designs.

Another limitation of this review is that a second reviewer only assessed 10% of the identified studies due to resource constraints. While this limited assessment may introduce bias, efforts were made to minimise this bias by selecting studies at random and holding regular discussions between reviewers and senior reviewers.

Although this study focuses on co-production, lay people or service users were not involved in the design, conduct, or interpretation of this systematic review. This reflects the nature of systematic reviews, which synthesises existing literature rather than generate new data. We felt the synthesis and analysis of previous literature would require previous academic training that we were unable to consider/provide for this review. However, many included studies incorporated lay perspectives, which are reflected in our analysis and findings. Importantly, the insights gathered from this review inform the design of a follow-up coproduction study currently being conducted by our team, which directly involves individuals with lived experience as core members of the research team. That on-going study has been shaped by the structure and outcomes of this review. Looking ahead, we also recommend that future work consider developing training for lay contributors to support meaningful involvement in systematic review processes.

## Conclusion

This review highlights the need to meaningfully involve affected loved ones in co-production for mental health and substance use resources/interventions throughout all stages of the research process. Three key strategies - building trust, fostering accessibility, and integrating diverse perspectives - are essential to support a rich and engaging co-production environment.

Our findings reveal that while co-production can be powerful, inconsistencies in strategy, terminology, and reporting hinder its full potential. To move the field forward, we need standardised definitions and global guidelines that ensure consistency and enhance the reliability of co-production approaches.

A major gap remains: the lack of co-production studies focused on substance use interventions with affected loved ones. Our group is currently conducting a study addressing this gap and following the proposed principles mentioned throughout this review. Addressing this will help create more inclusive, evidence-based solutions that can potentially help save lives.

By addressing these gaps and prioritizing inclusivity, future research can develop interventions that genuinely reflect the needs and voices of affected loved ones, driving more holistic and impactful solutions to the global mental health and substance use crisis.

## Electronic supplementary material

Below is the link to the electronic supplementary material.

Supplementary Material 1

Supplementary Material 2

Supplementary Material 3

## Data Availability

No datasets were generated or analysed during the current study.

## Abbreviations

DSM-5: Diagnostic and Statistical Manual of Mental Disorders, 5th Edition
WHO: World Health Organisation
PCIE: Public and Community Involvement and Engagement
MMAT: Mixed Methods Appraisal Tool
PRISMA: Preferred Reporting Items for Systematic Reviews and Meta-Analyses
RTA: Reflexive Thematic Analysis
RCA: Relational Content Analysis
GUIDED: Guidance for Reporting Intervention Development Studies
GRIPP2: Guidance for Reporting Involvement of Patients and the Public
NIHR: National Institute for Health and Care Research
