# Supplementary material for: A systematic review of co-production approaches that involve family members, loved ones, or carers in the development of mental health or substance use resources/interventions
Source: Res Involv Engagem. 2025 Oct 16;11:119. doi: 10.1186/s40900-025-00758-4 (PMC12532869; doi:10.1186/s40900-025-00758-4)
Supplement: Supplementary file 1 — Supplementary Material 1 [file 40900_2025_758_MOESM1_ESM.docx]

# Search Strategy:

The search will be carried out on these databases: Cochrane Central Register of Controlled Trials (CENTRAL), MEDLINE, Open Grey, ProQuest Thesis and Dissertations, PSYCInfo, Web of Science, and Scopus. A secondary search will be done on Google Scholar and relevant articles on the first 15 pages of the results will be included in the screening. The relevant keywords must be included in the title or abstract to be retrieved. A librarian (MS) supported the reviewer in deciding the databases and keywords with reference to the MeSH database. The reviewer will adjust the search terms for each data base. The reviewer will check the reference lists of eligible studies to identify other potential eligible studies to be included.

# Keywords:

Search in Title/Abstract

famil* OR mother* OR father* OR parent* OR sibling* OR sister* OR brother* OR grandparent* OR child* OR son* OR daughter* OR spouse* OR wife OR husband* OR wives OR “loved one*” OR partner* OR friend* OR carer* OR caregiver* OR caretaker* OR “care giver*” OR “care taker*” OR guardian* OR relative* OR “significant other*” OR “primary supporter” OR “informal caregiver”

AND

Search in Title/Abstract

Co-produ* OR co-creat* OR co-design* OR co-develop* OR coprodu* OR cocreat* OR codesign* OR codevelop* OR co-evaluat* OR coevaluat* OR co-implement* OR coimplement*

AND

Search in Title/Abstract

“mental health*” OR “mental well-being” OR “psychological well-being” OR “emotional well-being” OR “behavioural health” OR “behavioral health” OR “behavioural disorder*” OR “behavioral disorder*” OR “emotional disorder*” OR “anxiety disorder*” OR “personality disorder*” OR “ depressive disorder*” OR depress* OR “neurodevelopmental disorder*” OR addiction* OR “substance-use*” OR “substance use*” OR SUD OR “use disorder” OR misuse OR dependen* OR “substance abus*” OR “ substance dependen*” OR “drug dependen*” OR “substance related disorder*” OR rehab* OR therap* OR “psychosocial intervention*” OR “behavioural intervention*” OR “behavioral intervention” OR “treatment program*” OR “treatment service*” OR “recovery program*” OR “recovery service*” OR detox*

## Google Scholar Keywords:

family|”family member”|mother|father|parent|sibling|child|sister|brother|grandparent|son|daughter|spouse|wife|husband|wives|guardian|relative|”significant other”|”primary supporter|”loved one|carer|caregiver|caretaker|”care taker”|”care giver”|”informal caregiver” co-production|co-creation|co-design|co-developed|coproduction|cocreation|codesign|codevelopment|co-implement|coimplement “mental health”|”mental well-being”|”behavioural disorder”|”behavioral disorder”|addiction|substance-use|”substance use disorder”|”drug dependence”|”substance related”|treatment|support|recovery|program|therapy|intervention|depression|”emotional disorder”|”anxiety disorder”|”personality disorder”|”depressive disorder”|”neurodevelopmental disorder”|SUD|”use disorder”|misuse|depence|”psychosocial intervention”|detox
