# Supplementary material for: A systematic review of co-production approaches that involve family members, loved ones, or carers in the development of mental health or substance use resources/interventions
Source: Res Involv Engagem. 2025 Oct 16;11:119. doi: 10.1186/s40900-025-00758-4 (PMC12532869; doi:10.1186/s40900-025-00758-4)
Supplement: Supplementary file 2 — Supplementary Material 2 [file 40900_2025_758_MOESM2_ESM.docx]

| First author, year | MMAT Rating | MMAT Comment | Sufficiency of reporting of the co-production checklist | | | | | | | Sufficiency of reporting rating |
| --- | --- | --- | --- | --- | --- | --- | --- | --- | --- | --- |
|  |  |  | Intention | Setting | Members | Intervention | Methods & Phases | Intensity & Schedule | Experiences Evaluated |  |
| Acton et al., 2022 | ♦♦♦♦♦ | All criteria met | Y | Y | Y | Y | L | L | N | █ █ █ █ █ |
| Brooks et al., 2021 | ♦♦♦♦♦ | All criteria met | N | Y | Y | Y | Y | L | N | █ █ █ █ ▄ |
| Brooks et al., 2022 | ♦♦♦♦♦ | All criteria met | Y | Y | L | Y | Y | L | N | █ █ █ █ █ |
| Cheng et al., 2024 | ♦♦♦ | 5.2, 5.3 not met | Y | L | Y | L | Y | Y | N | █ █ █ █ █ |
| Chivers et al., 2005 | ♦♦♦♦ | 5.3 can’t tell | Y | L | Y | Y | Y | L | Y | █ █ █ █ █ █ |
| Cullingham et al., 2024 | ♦♦♦♦♦ | All criteria met | Y | L | Y | Y | Y | Y | N | █ █ █ █ █ ▄ |
| Davies et al., 2016 | ♦♦ ♦ | 1.3 can’t tell, 1.4 not met | Y | L | L | Y | Y | N | N | █ █ █ █ |
| Dodd et al., 2022 | ♦♦♦ | 5.2, 5.5 not met | Y | L | Y | L | Y | Y | N | █ █ █ █ █ |
| Egan et al., 2023 | ♦♦♦♦♦ | All criteria met | Y | Y | Y | L | L | Y | N | █ █ █ █ █ |
| Goeman et al., 2017 | ♦♦♦♦♦ | All criteria met | Y | Y | L | L | Y | Y | N | █ █ █ █ █ |
| Hackett et al., 2018 | ♦♦♦♦♦ | All criteria met | Y | L | L | Y | Y | N | N | █ █ █ █ |
| Higgins et al., 2017 | ♦♦♦♦♦ | All criteria met | Y | Y | Y | Y | L | L | N | █ █ █ █ █ |
| Jerwood, 2019 | ♦♦♦♦♦ | All criteria met | Y | Y | L | Y | Y | L | N | █ █ █ █ █ |
| Kaur et al., 2024 | ♦♦♦♦♦ | All criteria met | Y | L | Y | Y | Y | Y | N | █ █ █ █ █ ▄ |
| Leadbitter et al., 2024 | ♦♦♦♦ | 5.1 not met | Y | Y | L | Y | Y | L | N | █ █ █ █ █ |
| Lopes et al., 2016 | ♦♦♦♦ | 1.4 not met | N | Y | Y | Y | Y | L | N | █ █ █ █ ▄ |
| Mbazzi et al., 2020 | ♦♦♦♦♦ | All criteria met | Y | L | L | L | L | L | Y | █ █ █ █ ▄ |
| McAllister et al., 2021 | ♦♦♦♦♦ | All criteria met | Y | Y | L | Y | Y | L | Y | █ █ █ █ █ █ |
| Middleton et al., 2022 | ♦♦♦ | 5.1, 5.5 not met | Y | Y | Y | Y | Y | L | Y | █ █ █ █ █ █ ▄ |
| Milton et al., 2021 | ♦♦♦♦♦ | All criteria met | Y | Y | Y | Y | Y | Y | N | █ █ █ █ █ █ |
| Molloy et al., 2024 | ♦♦♦♦♦ | All criteria met | Y | Y | L | Y | L | L | Y | █ █ █ █ █ ▄ |
| Mulvale et al., 2020 | ♦♦♦♦♦ | All criteria met | Y | Y | Y | Y | Y | Y | Y | █ █ █ █ █ █ █ |
| Murfield et al., 2022 | ♦♦♦♦♦ | All criteria met | Y | Y | Y | N | Y | Y | N | █ █ █ █ █ |
| Nakarada-Kordic et al., 2017 | ♦♦♦♦ | 5.3 not met | Y | Y | N | Y | Y | Y | N | █ █ █ █ █ |
| Oksnebjerg et al., 2019 | ♦♦♦♦♦ | All criteria met | Y | Y | Y | Y | Y | L | N | █ █ █ █ █ ▄ |
| Oostra et al., 2023 | ♦♦♦♦♦ | All criteria met | N | L | Y | Y | Y | Y | N | █ █ █ █ ▄ |
| Rapaport et al., 2018 | ♦♦♦♦ | 1.4 not met | Y | L | L | L | L | L | N | █ █ █ ▄ |
| Rathnayake et al., 2021 | ♦♦♦ | 5.1, 5.5 not met | Y | L | Y | Y | Y | L | N | █ █ █ █ █ |
| Rivard et al., 2024 | ♦♦♦♦♦ | All criteria met | Y | L | L | L | Y | L | Y | █ █ █ █ █ |
| Robinson et al., 2020 | ♦♦♦ | 5.1, 5.5 not met | Y | Y | Y | Y | Y | L | N | █ █ █ █ █ ▄ |
| Sin et al., 2019 | ♦♦♦♦♦ | All criteria met | Y | L | Y | Y | Y | L | N | █ █ █ █ █ |
| Tarver et al., 2021 | ♦♦ | 5.3, 5.5 not met, 5.4 can’t tell | N | L | Y | Y | Y | L | N | █ █ █ █ |
| Turuba et al., 2024 | ♦♦♦♦ | 5.2 not met | Y | Y | Y | L | Y | L | Y | █ █ █ █ █ █ |
| Ung et al., 2023 | ♦♦♦♦ | 5.1 not met | Y | L | L | Y | L | L | N | █ █ █ █ |
| Vijayalakshmi et al., 2024 | ♦♦♦♦♦ | All criteria met | Y | Y | L | Y | Y | L | N | █ █ █ █ █ |
| Wittich et al., 2023 | ♦♦♦♦ | 1.4 not met | Y | Y | Y | L | Y | Y | N | █ █ █ █ █ ▄ |
| Wormdahl et al., 2022 | ♦♦♦♦ | 1.4 not met | Y | Y | L | Y | L | Y | N | █ █ █ █ █ |
| Wood et al., 2023 | ♦♦♦ | 1.4 not met, 1.5 can’t tell | Y | Y | Y | L | Y | Y | N | █ █ █ █ █ ▄ |
| Zervogianni et al., 2020 | ♦♦♦♦♦ | All criteria met | Y | Y | Y | Y | Y | Y | N | █ █ █ █ █ █ |
| Zhu et al., 2024 | ♦♦♦♦♦ | All criteria met | Y | Y | L | Y | Y | Y | N | █ █ █ █ █ ▄ |

Table X. Quality of Studies based on the MMAT and sufficiency of reporting of the co-production checklist.
