# Supplementary material for: A systematic review of co-production approaches that involve family members, loved ones, or carers in the development of mental health or substance use resources/interventions
Source: Res Involv Engagem. 2025 Oct 16;11:119. doi: 10.1186/s40900-025-00758-4 (PMC12532869; doi:10.1186/s40900-025-00758-4)
Supplement: Supplementary file 3 — Supplementary Material 3 [file 40900_2025_758_MOESM3_ESM.docx]

| Author, year | Country | Study Design | Term used for co-production approach | Type of Mental Health/Substance Use concern | Developed/adapted resource/intervention |
| --- | --- | --- | --- | --- | --- |
| Acton et al., 2022 | UK | Qualitative | Co-production | Intellectual Disabilities, Dementia | Cognitive stimulation therapy manual |
| Brooks et al., 2021 | Indonesia | Qualitative | Co-development | Youth mental health | Storyline digital application |
| Brooks et al., 2022 | UK | Qualitative | Co-production | Mental health | Social network intervention |
| Cheng et al., 2024 | USA | Mixed Methods | Co-production | Psychosis | Cognitive behavioural therapy digital platform |
| Chivers, 2005 | UK | Qualitative | Co-creation | Severe communication difficulties | Practice framework and model for service development |
| Cullingham et al., 2024 | UK | Qualitative | Co-Design | Autism and anxiety | Adapted an online interactive multi-modal module to support anxiety |
| Davies et al., 2016 | UK | Qualitative | Co-design | Dementia | Heuristic toolkit |
| Dodd et al., 2022 | UK | Mixed Methods | Co-production | Dementia | Nostalgia intervention |
| Egan et al., 2023 | Australia | Mixed Methods | Co-design | Adolescent eating disorders | Parent-supported CBT-P-ED |
| Goeman et al., 2017 | Australia | Qualitative | Co-development | Dementia | Discussion tool |
| Hackett et al., 2018 | Canada | Qualitative | Co-design | Youth mental health | Prototype Questionnaire |
| Higgins et al., 2017 | Ireland | Qualitative | Co-production | Schizophrenia/Bipolar disorder | Mental Health Information Programme |
| Jerwood, 2019 | UK | Qualitative | Co-design | Severe mental illness | Website |
| Kaur et al., 2024 | USA | Mixed Methods | Co-Design | Autism | Developed app for challenging behaviours and emotional dysregulation |
| Leadbitter et al., 2024 | UK | Mixed Methods | Co-Design | Autism | Developed psychoeducation and psychotherapeutic programme for caregivers |
| Lopes et al., 2016 | France | Qualitative | Co-design | Cognitive impairment | Assistive device to track items |
| Mbazzi et al., 2020 | Uganda | Qualitative | Co-design | Intellectual and neurological disabilities | Peer-to-peer intervention |
| McAllister et al., 2021 | UK | Qualitative | Co-development | Severe mental illness | Engagement Intervention Toolkit |
| Middleton et al., 2023 | Canada | Mixed Methods | Co-development | Dementia | Toolkit for physical activity |
| Milton et al., 2021 | Australia | Qualitative | Co-design | Eating disorders and disordered eating | Web based information and communication platform |
| Molloy et al., 2024 | Australia | Qualitative | Co-design | Mental health | Health nursing curriculum |
| Mulvale et al., 2020 | Canada | Qualitative | Co-design | Mental health | Hospital transition prototype |
| Murfield et al., 2022 | Australia | Qualitative | Co-design | Dementia | Self-compassion intervention |
| Nakarada-Kordic et al., 2017 | UK | Qualitative | Co-design | Youth psychosis | Education and wellbeing webpage |
| Oksnebjerg et al., 2019 | Denmark | Qualitative | Co-design | Dementia | App for self-management |
| Oostra et al., 2023 | The Netherlands | Qualitative | Co-design | Dementia | Digital resilience monitor |
| Rapaport et al., 2018 | UK | Qualitative | Co-production | Dementia | Sleep intervention |
| Rathnayake et al., 2021 | Australia | Mixed Methods | Co-production | Dementia | Mobile health application for care needs |
| Rivard et al., 2024 | Canada | Qualitative | Co-development | Child developmental disabilities | Assessment and support service trajectory |
| Robinson et al., 2020 | UK | Mixed Methods | Co-design | Child learning disability |  |
| Sin et al., 2019 | UK | Qualitative | Co-production | Psychosis | Electronic health intervention |
| Tarver et al., 2021 | UK | Mixed Methods | Co-development | Autism | Assessment questionnaire |
| Turuba et al., 2024 | Canada | Mixed Methods | Co-design | Youth opioid use disorders | Handbook |
| Ung et al., 2023 | Australia | Mixed Methods | Co-design | Psychosis | Stimulated patient scenarios |
| Vijayalakshmi et al., 2024 | India | Qualitative | Co-design | Psychosis | Exercise videos |
| Wittich et al., 2023 | Canada | Qualitative | Co-design | Dementia | Web-based multidomain literacy program |
| Wood et al., 2023 | UK | Qualitative | Co-production | Psychosis | Cognitive behavioural therapy for psychosis |
| Wormdahl et al., 2022 | Norway | Qualitative | Co-creation | Severe mental illness | Reducing coercion intervention |
| Zervogianni et al., 2020 | UK | Mixed Methods | Co-development | Autism | Framework for digital supports |
| Zhu et al., 2024 | China | Mixed Methods | Co-design | Mild cognitive impairment | Digital storytelling intervention |
